# 1345. Patient Perspectives and Journey with Influenza and Seeking Care from US National Survey

**DOI:** 10.1093/ofid/ofab466.1537

**Published:** 2021-12-04

**Authors:** Nate Way, Ashley Martin, Chris Wallick, Edward Neuberger, Mitra Corral

**Affiliations:** 1 Health Outcomes Practice, Kantar Health, San Mateo, California; 2 Genentech, Inc., South San Francisco, CA

## Abstract

**Background:**

In the 2017-2018 influenza season, 49 million people in the US presented with influenza symptoms, resulting in substantial morbidity, mortality, and a significant humanistic and economic burden. Although there are currently four FDA-approved antivirals for influenza, such treatments continue to be widely underutilized. The aim of this study was to better understand the patients’ perspective and experience with a flu episode and seeking care during the 2019-2020 influenza season.

**Methods:**

Data were obtained from an online quantitative survey of influenza patients. Participants were recruited from two data sources: A pool of respondents who previously completed the National Health and Wellness Survey (NHWS) (N=74,977) or from Lightspeed M3 Global online “General Panel” (N=500,000+) in the US from January 2020 through May 2020. The sample included patients >18 years of age and having a self-reported diagnosis of influenza by a healthcare professional within the last 90 days. Outcomes related to patient demographics, health-related characteristics and perspectives on the influenza episode were collected.

**Results:**

1,005 patients were included. Of those, 30.2% visited their primary health care professional (HCP) in person, 20.2% visited urgent care walk-in facility and 19.2% called their HCP. Important aspects of flu treatment included: feeling better quickly (69.5%), not transmitting to others (51.5%), and ease of administration (40.7%); 375 patients were treated with an antiviral. Of those, it took~4.6 days to feel generally better and ~8.8 days to feeling totally better. About 73% of patients took all of their antiviral medication, 9% took “some”. 43.9% of respondents considered themselves to be more likely to get serious flu-related complications, 52.5% reported that they were told by an HCP that they belong to a high-risk group that may be more likely to get flu-related complications. 41.3% reported they did not experience any high risk factors while experiencing influenza.

**Conclusion:**

Influenza patients reported different attitudes and treatment approaches to handling their infection. It is critical to understand what matters most to patients regarding both influenza and treatment to optimally provide outreach and care.

**Disclosures:**

**Nate Way, PhD**, **Genentech, Inc.** (Grant/Research Support)**Kantar Health** (Employee) **Ashley Martin, PhD**, **Genentech, Inc.** (Grant/Research Support)**Kantar Health** (Employee) **Chris Wallick, PharmD, MS**, **Genentech, Inc.** (Employee, Shareholder) **Edward Neuberger, PharmD, MBA, MS**, **Genentech, Inc.** (Employee, Shareholder) **Mitra Corral, MS, MPH**, **Genentech, Inc.** (Employee, Shareholder)

